# Microencapsulation of Extracts of Strawberry (*Fragaria vesca*) By-Products by Spray-Drying Using Individual and Binary/Ternary Blends of Biopolymers

**DOI:** 10.3390/molecules29194528

**Published:** 2024-09-24

**Authors:** Yara Bastos, Fernando Rocha, Berta Nogueiro Estevinho

**Affiliations:** 1 LEPABE—Laboratory for Process Engineering, Environment, Biotechnology and Energy, Department of Chemical Engineer, Faculty of Engineer, University of Porto, Rua Dr. Roberto Frias, 4200-465 Porto, Portugal; 2 ALiCE—Associate Laboratory in Chemical Engineer, Faculty of Engineering, University of Porto, Rua Dr. Roberto Frias, 4200-465 Porto, Portugal

**Keywords:** add-value products, agri-food waste, antioxidants, circular economy, cosmetic application, food products, quercetin, microparticles, polyphenols

## Abstract

Valorization of agricultural and food by-products (agri-food waste) and maximum utilization of this raw material constitute a highly relevant topic worldwide. Agri-food waste contains different types of phytochemical compounds such as polyphenols, that display a set of biological properties, including anti-inflammatory, chemo-preventive, and immune-stimulating effects. In this work, the microencapsulation of strawberry (*Fragaria vesca*) plant extract was made by spray-drying using individual biopolymers, as well as binary and ternary blends of pectin, alginate, and carrageenan. The microparticle morphologies depended on the formulation used, and they had an average size between 0.01 μm and 16.3 μm considering a volume size distribution. The encapsulation efficiency ranged between 81 and 100%. The kinetic models of Korsmeyer–Peppas (R^2^: 0.35–0.94) and Baker–Lonsdale (R^2^: 0.73–1.0) were fitted to the experimental release profiles. In general, the releases followed a “Fickian Diffusion” mechanism, with total release times varying between 100 and 350 (ternary blends) seconds. The microparticles containing only quercetin (one of the main polyphenols in the plant) showed higher antioxidant power compared to the extract and empty particles. Finally, the addition of the different types of microparticles to the gelatine (2.7 mPa.s) and to the aloe vera gel (640 mPa.s) provoked small changes in the viscosity of the final gelatine (2.3 and 3.3 mPa.s) and of the aloe vera gel (621–653 mPa.s). At a visual level, it is possible to conclude that in the gelatine matrix, there was a slight variation in color, while in the aloe vera gel, no changes were registered. In conclusion, these microparticles present promising characteristics for food, nutraceutical, and cosmetic applications.

## 1. Introduction

The consumption of fruits and vegetables is largely widespread throughout the world, being the basis of most diets, not only due to their nutritional characteristics, but also due to their nutraceutical and health potential [1,2,3]. Furthermore, the use of raw materials such as flowers, fruits, leaves, seeds, roots, skins, and herbs are frequently used to obtain extracts for nutraceutical, cosmetic, and pharmaceutical preparations [1,2,3]. For example, curcumin [4,5,6] and resveratrol [7,8,9] are well-known examples of antioxidant compounds extracted from plants and fruits and they are used in several applications.

Wild strawberry, scientifically known as *Fragaria vesca*, is a member of the Rosacea family and is usually found in Europe, West Asia, and North America [10,11,12,13]. According to the data of FAO (2022), the world production of strawberries was 9.6 million tons, with China being responsible for 35% of the total production. Other countries like the USA, Turkey, Egypt, and Mexico are also in the group of the main producers. However, it is reported that more acres of strawberries (world’s 19th most popular fruit) are necessary to fill the growing worldwide demand. This plant has a rich history in traditional medicine, where various beneficial biological effects of strawberry fruit consumption have been documented, such as an increase in the serum antioxidant capacity in humans, anti-carcinogenic activity, anti-thrombotic effects, anti-inflammatory properties, and its potential in the treatment of gastrointestinal disorders [10,11,12,13,14,15,16]. These beneficial effects have been mostly attributed to the phenolic compounds [13,17,18,19]. The main phenolic compounds present in this plant are flavonoids, ellagitannins, proanthocyanidins, phenolic acids, catechins, volatile oils, methyl salicylate, ellagic acid, borneol, and also trace amounts of alkaloids [1,11,18,20,21,22,23].

Normally in the agricultural process, the leaves of *F. vesca* are thrown away and treated as waste. Nevertheless, they can be a rich source of valuable, biologically active substances with a wide range of applications in the preparation of food/nutraceutical, cosmetic, and pharmaceutical formulas [1,2,21,24]. For example, Mudnic et al. (2009) studied the vasodilatory potential of the wild strawberry leaves extract that could be associated with the extracts rich in the flavonoid monomers catechin and epicatechin [13]. The strawberry leaves are described as a source of important bioactive compounds such as ellagitannins, proanthocyanidins, quercetin, and kaempferol glucuronide derivatives, and more precisely, procyanidin B1, B2, and C1, pyrocyanidin B1, epigallocatechin, (+)-catechin, (−)-epicatechin, astringin, epicatechin-3- gallate, piceid, quercetin, quercetin-4’-glucoside, gallic acid monohydrate, kaempferol 3-β-d-glucopyranoside, and trans-resveratrol [25,26]. The leaves of *F. vesca* extracts have potential beneficial and biological effects and have been largely used in traditional medicine for the treatment of several diseases, namely to help the digestive, respiratory, nervous, and female reproductive systems [13,21,25].

Therefore, in this work, the by-products from wild strawberries were used to prepare innovative add-value formulations to be applied in different fields such as food, nutraceuticals, and cosmetics, considering their health benefits [15]. The valorization of by-products, namely agricultural and food by-products (agri-food waste), are one of the goals of the United Nations for 2030 to reduce food waste production, to achieve a more sustainable world [27]. Agro-food activities are characterized by high amounts of organic waste, which can represent more than 50% of the fresh fruit [21,28,29].

Agri-food wastes can be the sources of innumerous value-added products, and this should be the way to achieve a zero-waste economy [30,31,32]. The circular economy applied to the agro-food activities promotes new strategies to finish with environmental problems, and at the same time, contribute to obtain value from this type of waste [33,34].

Therefore, *F. vesca* by-product extracts can present several health benefits if correctly incorporated into foods, nutraceuticals, or cosmetic products [11]. Most of the bioactive compounds extracted are very sensitive compounds that need to be protected from external environmental conditions like oxygen, light, or temperature, to keep their biological properties. Microencapsulation makes it possible to create a physical barrier between a sensitive compound, like the phenolic compounds of the extract, and the external factors [21,35]. One of the most popular encapsulation techniques in the food and pharmaceutical industry is spray-drying [35].

Spray-drying is an effective method to improve the stability of bioactive substances against oxidation and degradation, and to produce powdered formulations. The powdered products produced are more convenient and safer to transport, store, and process than other liquid formulations [36,37].

On the other hand, biopolymeric microparticles, thanks to their extraordinary biological compatibility and natural origin, have been widely used and studied for the encapsulation and delivery of functional compounds in nutraceutical/food and cosmetic products. Numerous wall materials are available for the encapsulation of bioactive compounds, including carbohydrates, proteins, and lipids, with special attention being given to carbohydrates, due to their biodegradability, biocompatibility, low cost, diversity, and good structural support ability. Furthermore, in comparison with the other encapsulating agents, carbohydrates may provide protection against higher temperatures and, because of their widespread use in several industries, there is a high level of knowledge regarding their use [35,36,38].

So, in this work, it was proposed to produce microparticles to carry *F. vesca* by-product extracts by spray-drying. The microencapsulation process will be tested using individual biopolymers, binary and ternary blends of pectin, alginate, and carrageenan. Our goal was to create biocompatible and non-toxic microparticles with good properties in terms of encapsulation efficiency and controlled release. Pectin, alginate, and carrageenan have been extensively employed in diverse industries, including pharmaceuticals, cosmetics, food, and in delivery systems. They have received approval from the Food and Drug Administration (FDA) as generally recognized as safe products (GRAS). Pectin is a natural polysaccharide and can be found in fruits, namely in citrus fruits and in apples. It is a gelling agent and contributes to the solidification of jams. Pectin can be a promising encapsulation material due to its ability to act as an emulsion stabilizer and to its gelling and binding capabilities [36]. Alginate is a common polysaccharide used in delivery systems, being resistant to the acidic stomach environment, arising as a good solution for bioactive compound release in the intestine [35]. Carrageenan is a linear anionic heteropolysaccharide extracted from red algae and it is widely used in the food industry as a gelling, stabilizing, and thickening agent [24]. The microparticles obtained will be characterized and the encapsulation efficiency will be evaluated.

Therefore, the innovative and relevant point of this study is related to the utilization of agro-industrial by-products from *F. vesca* in add-value encapsulated formulations as food/cosmetic additives, allowing the promotion of the circular economy. To the best of our knowledge, no studies of microencapsulation have been performed with the extracts of the *F. vesca* by-products using a spray-drying method.

So, the primary aim of this research was to assess the microparticles’ characteristics produced using the natural *F. vesca* by-products extract and quercetin (one of the main polyphenols in the plant), and to compare the morphology, size, and release properties of the different microparticles obtained with different polymer formulations. Quercetin is widely used in cosmetics, nutraceuticals, and pharmaceuticals due to its high antioxidant and anti-inflammatory potential. Additionally, the antioxidant activity of the microparticles was evaluated. Finally, preliminary tests of microparticles’ incorporation in food and cosmetic commercial products were made and the impact of the use of these additives in these types of products was evaluated.

## 2. Results and Discussion

The main objective of this study was to produce microstructures containing quercetin and strawberry leaves extract, with high encapsulation efficiency, using a spray-drying technique, and to evaluate their potential for application in the food and cosmetic industries.

Therefore, three different encapsulating agents (alginate (Alg), carrageenan (Carr), and pectin (Pec)) were tested to produce the microparticles. The encapsulation effectiveness of each polymer, both individually and in binary and ternary mixtures, was evaluated. The same experimental design was followed for all the formulations. The microparticles obtained were characterized by size, morphology, and release profiles. The antioxidant capacity of the microparticles and the encapsulation efficiency were evaluated. Finally, preliminary studies of food and cosmetic incorporation of the microparticles were conducted.

### 2.1. Characterization of the Microparticles

**Figure 1:**
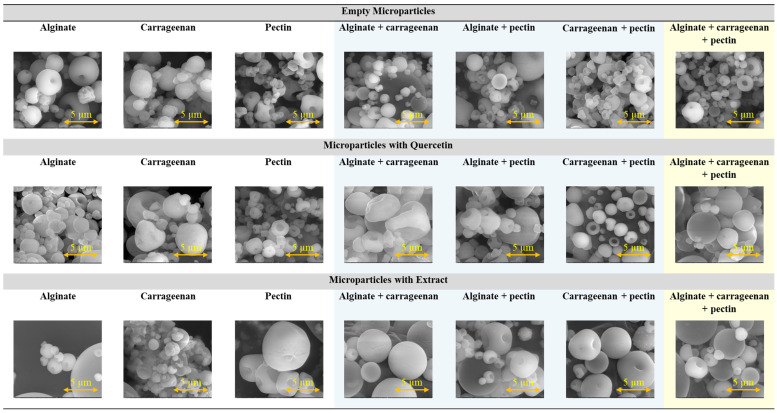
SEM images of microparticles prepared by spray-drying with different encapsulating agents (alginate, pectin, and carrageenan) considering individual and binary/ternary blends. Empty microparticles and microparticles containing *F. vesca* leaves extract and quercetin were considered. Magnification of 20,000×, a beam intensity (HV) of 15.00 kV, and a distance between the sample and the lens (WD) of less than 11 mm (bar in the images = 5 µm).

Microparticles containing *F. vesca* leaves extract and quercetin were evaluated by SEM (Figure 1). The images acquired were important to evaluate the shape, morphology, and size of the microparticles, as well as the size distribution and aggregation of particles, which are essential factors to consider when considering industrial applications.

For all the formulations tested, spherical microparticles with a regular shape were produced (Figure 1). In terms of size, it is possible to verify that all samples have a heterogeneous size distribution, typical of the spray-drying process, with some small particles located among bigger particles. In general, the microparticles had diameters around 1–5 µm.

The surface of the microparticles presented textural characteristics depending on the encapsulating agent used, being specific for each of them. Alginate particles show a spherical structure, with a smooth surface, and a slightly porous surface. Pectin particles show a spherical structure with a rough or porous surface. Carrageenan particles have an irregular surface with a granular and slightly porous appearance. These results are in accordance with the literature and with previous studies of the authors, considering a spray-drying methodology and this type of encapsulating agent [35,36,39]. The microparticles prepared by spray-drying usually show a spherical shape, with a low average particle size ranging from 5 to 50 µm, and have a smooth outer surface or exhibit the formation of teeth or concavities with an irregular shape [39,40,41].

In general, the physical properties of the microparticles are similar regardless of the presence of the bioactive compound, and the encapsulating agent is the key determinant of these properties. Considering the SEM images, the size of the microparticles containing extract appear to be bigger than the empty microparticles and the ones containing quercetin. For the case of the ternary blend of encapsulating agents, the microparticles containing bioactive compound, namely the ones containing extract, are slightly bigger than the empty microparticles.

The size of the microparticles was confirmed in complementary studies performed by laser granulometry.

### 2.2. Size Distribution of the Microparticles

Microparticles containing *F. vesca* leaves extract and quercetin were evaluated in terms of particle size distribution (Table 1). This information can be useful to understand the behavior of the microparticles in relation to the controlled release of the encapsulated compound and to infer about the stability of them. The particle size can influence the texture and sensory properties of the final product [35,42]. In general, the size homogeneity is important for industrial applications.

According to the results, the size of microparticles considering a volume differential distribution varied between 0.1 µm and 16.3 µm. In terms of the number differential distribution, the values varied between 0.06 µm and 1.00 µm. Therefore, different values for the average particle size were obtained considering the different distributions (number and volume) which may suggest some aggregation. The spray-drying method produced microparticles with some size diversity, which is in accordance with other studies and with the literature [35,36].

To the best of our knowledge, there are no studies of microencapsulation of the strawberry leaves extract by spray-drying. However, there are some studies of microencapsulation of polyphenols or extract from strawberry fruits. Pulicharla et al. (2016) studied the encapsulation and release of strawberry polyphenols in biodegradable chitosan nanoformulations, and they formed particles with a size that ranged between 300 and 600 nm [43]. Also, Tatar et al. (2019) studied the microencapsulation of phenolic powder obtained from strawberry pomace using different combinations of maltodextrin and gum Arabic [44]. In this study, the microparticles have average size ranging from 4 to 63 µm, depending on the operational conditions used. The encapsulation of strawberry flavor was made by Balci-Torun and Ozdemir (2021) using a spray-drying process and the particles exhibited a wide range of diameters, ranging from 0.32 μm to about 69.25 μm [45]. The microencapsulation of strawberry juice in *Agave angustifolia* fructans was performed by a spray-drying process, with particles presenting some heterogeneity in terms of size, being the biggest (3.5 ± 2.2 μm) spherical hollow microcapsules [46]. Finally, Baldelli et al. (2024) studied the microencapsulation of common fruit juices, namely strawberry, by spray-drying using different biopolymers as encapsulating agents, namely hydroxypropyl methylcellulose (HPMC) and whey protein, obtaining microparticles between 80 and 300 µm [47].

### 2.3. Conductivity and Viscosity of Biopolymer Solutions Used to Prepare the Microparticles

These two parameters are important considering a mechanical encapsulation process, such as spray-drying, and the type of industrial application of the microparticles. The viscosity has extreme relevance considering the operational restrictions related to the viscosity of the samples fed to the spray-dryer. The biopolymer solutions (individual and binary/ternary blends of biopolymers) were characterized in terms of electrical conductivity and viscosity; the results are shown in Table 2.

In terms of conductivity, it was possible to conclude that carrageenan is the encapsulating agent that presents higher conductivity, helping to increase the conductivity of binary solutions where it was present. The biopolymer with the lowest conductivity was pectin, with almost zero conductivity.

In terms of viscosity, alginate solution had a very low viscosity compared to the other solutions. The biopolymer with the highest viscosity was carrageenan; therefore, it could be observed that solutions that contained carrageenan in their composition had higher viscosity. On an industrial scale, the solutions containing carrageen can imply some extra care or even some restrictions in terms of concentrations used.

### 2.4. Encapsulation Efficiency and In Vitro Release Studies

The encapsulation efficiency is one of the most relevant parameters to evaluate the quality of the microencapsulation of active compounds, indicating the amount of substances (bioactive compounds) that are successfully retained within the particles.

The encapsulation efficiencies of the *F. vesca* extract and quercetin are presented in Table 3.

Encapsulation efficiencies varied between 77 and 99% and it was possible to conclude that high encapsulation efficiencies were achieved. In general, the encapsulation efficiencies were higher for the extract.

The binary mixtures of encapsulating agents, namely the ones with alginate, presented a higher encapsulation efficiency than using only the individual agents. The ternary blend presented a higher efficiency to encapsulate the extract than the pure compound (quercetin).

**Figure 2:**
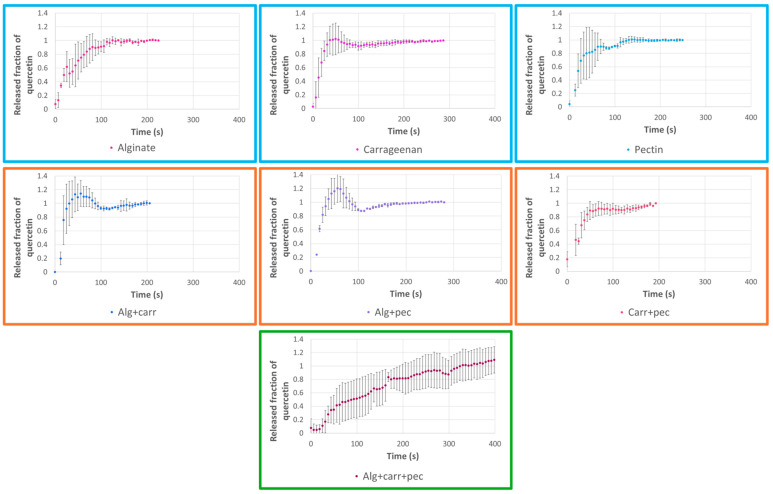
Release profiles of microparticles loaded with quercetin and prepared with different encapsulating agents (alginate, pectin, and carrageenan) considering individual and binary/ternary blends of them.

**Figure 3:**
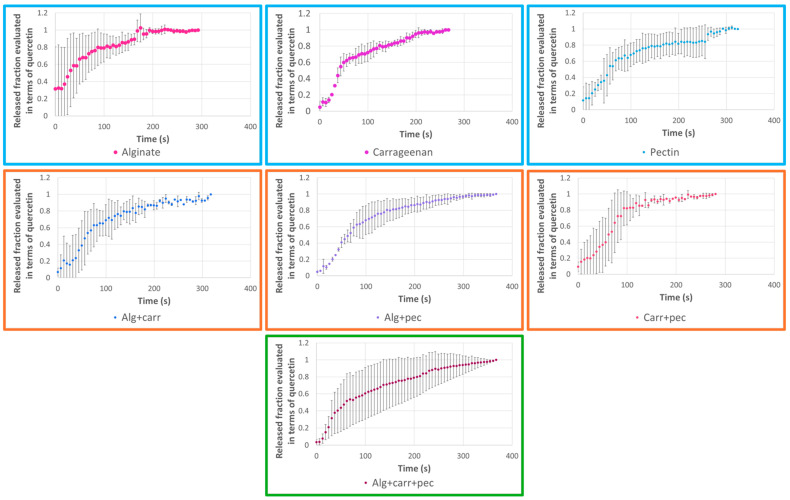
Release profiles of microparticles loaded with *F. vesca* leaves extract and prepared with different encapsulating agents (alginate, pectin, and carrageenan) considering individual and binary/ternary blends of them.

The release profiles were analyzed for each type of microparticles prepared containing quercetin (Figure 2) or strawberry leaves extract (using quercetin wavelength as an indicator) (Figure 3). The release profiles obtained with the microparticles containing quercetin (Figure 2) showed a fast release of the quercetin from the microparticles in general less than 100 s. The microparticles prepared with individual or binary blends presented a fast release. The microparticles prepared with the ternary blend presented the slowest release (350 s). In the case of the microparticles containing the extract, the release was slower than for the microparticles with quercetin. The releases profiles had a similar tendency allowing a release ranging from 100 to 350 s (Figure 3). The formulation prepared with the ternary blend also presented the slowest release of 350 s.

Finally, the release profiles were analyzed according to the Korsmeyer–Peppas kinetic model (Table 4). The Korsmeyer–Peppas model is a kinetic model widely used to study the release kinetics of drugs and other substances from controlled release systems [48]. The adjustment was performed with correlation coefficients ranging from 0.62 to 0.85 and from 0.35 to 0.94 for the quercetin microparticles and for the extract microparticles, respectively. The correlation coefficients were not very high considering that the release profiles presented some instability and “burst” in the beginning of the release. According to the value of the parameter n of the Korsmeyer–Peppas equation, it was possible to identify the major mechanism responsible for controlled release. The release exponent (n) was n < 0.43 for almost all the cases, which corresponds to a Fickian diffusion (case I transport). Two exceptions were observed for the microparticles prepared with binary blends and containing quercetin (Alg + carr and Alg + pec). In these two exceptions, I was observed 0.43 < n < 0.85, so the quercetin transport mechanism was by an anomalous transport that occurs involving both Fickian diffusion and polymer chain relaxation [48,49,50].

The Baker–Lonsdale model was also applied (Table 5). This model clarifies the controlled release kinetics of spherical monolithic dispersions, which is the case of the microparticles prepared by spray-drying and presents coefficients of correlation ranging from 0.73 to 1.0. The microparticles prepared with extract present higher correlation coefficients (with the exception of the sample of the microparticles prepared with the binary mixture carrageenan + pectin (0.84)) than the ones prepared with quercetin. The microparticles prepared with the ternary mixture of encapsulating agents presented the highest correlation coefficients for extract (1.0) and for the quercetin (0.98).

### 2.5. Antioxidant Activity

The antioxidant activity of the microparticles was evaluated using the ABTS method (Table 6). It is possible to evaluate the antioxidant power of the samples prepared by taking into account that the higher values of equivalent of micromoles of TROLOX are associated to higher antioxidant activity [21,51].

According to the results obtained in Table 6, it was possible to observe, as expected, that the microparticles containing only the encapsulating agent, present low values of equivalent of micromoles of TROLOX. Particles containing quercetin had high values of antioxidant power, corresponding to the highest values of equivalent of micromoles of TROLOX. The highest antioxidant power was associated with the carrageenan microparticles for the ones containing quercetin and also for the ones containing extract.

### 2.6. Preliminary Studies of Food Incorporation

Microparticles with interesting properties can be used in the food industry, in the incorporation into food products. According to the literature, consuming more than 650 mg/day of polyphenols reduce the risks of cardiovascular, carcinogenic, and inflammatory diseases [52,53]. However, this fortification and/or supplementation should be conducted with moderation [54,55].

In this study, a gelatine fortification was created by adding the microparticles into a commercial pineapple-flavored gelatine (powder formulation), in order to obtain a quantity of phenolic compound of 20.4 mg per 250 mL of gelatine.

**Figure 4:**
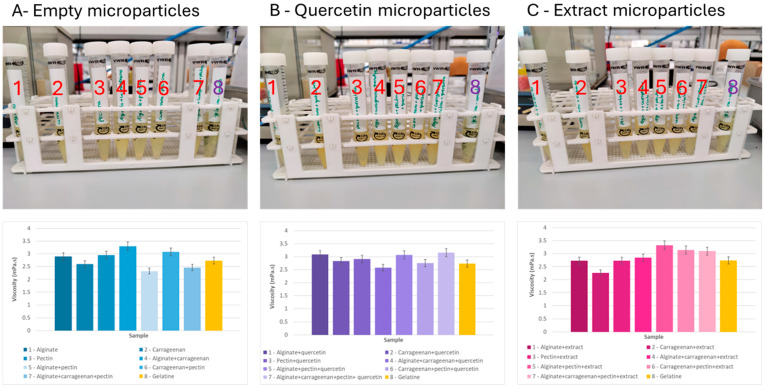
Gelatine (8) fortified with different formulations of microparticles: empty microparticles and loaded with quercetin and *F. vesca* leaves extract and prepared with different encapsulating agents (alginate—1, pectin—2, and carrageenan—3) considering individual and binary/ternary blends of them. (4—alginate + carrageenan, 5—alginate + pectin, 6—carrageenan + pectin, 7—alginate + carrageenan + pectin).

The different gelatine samples were evaluated in terms of visual appearance and viscosity (Figure 4).

In general, the fortified gelatine solutions containing the microparticles of quercetin or extract were opaquer compared to the control (only gelatine).

The empty microparticles and microparticles loaded with the extract did not change the color of the gelatine. The microparticles containing quercetin colored the gelatine with a more intense yellow color, because quercetin has a yellowish color.

For all the gelatine formulations containing microparticles prepared with different encapsulating agents, it was possible to observe that the gelatine solutions containing the quercetin particles were the ones with a darker yellow tone. It was also possible to observe that all solutions underwent a change in relation to transparency, becoming opaquer than the control solution (only gelatine).

Analyzing the results of viscosity (room temperature), the fortification of the gelatine (2.7 mPa.s) with the addition of the different types of microparticles provoked small changes in the viscosity of the final gelatine ranging between 2.3 and 3.3 mPa.s (empty microparticles: 2.5–3.3 mPa.s; quercetin microparticles: 2.6–3.2 mPa.s; extract microparticles: 2.3–3.3 mPa.s).

On the other hand, for the same type of microparticles prepared with the same encapsulating agent, the inclusion of quercetin or extract had different effects on the viscosity of the fortified gelatine. For example, gelatine fortified with empty microparticles prepared with a binary blend of alginate and carrageenan had a viscosity (3.3 mPa.s) higher than the gelatine without fortification (2.7 mPa.s). If these microparticles included quercetin, the viscosity (2.6 mPa.s) was reduced to values lower than gelatine without fortification. However, if the extract was incorporated instead of quercetin, the viscosity value (2.8 mPa.s) was higher than the gelatine without fortification.

The viscosity of gelatine solutions increased with increasing gelatine concentration and with decreasing temperature. According to the literature, the values of commercial gelatine range between 1 and 20 mPa.s [56,57,58].

Finally, it was possible to conclude that the viscosity of the gelatine did not change significantly with the addition of microparticles and that the values obtained were completely acceptable compared with the values of the commercial gelatines.

### 2.7. Preliminary Studies of Cosmetic Incorporation

**Figure 5:**
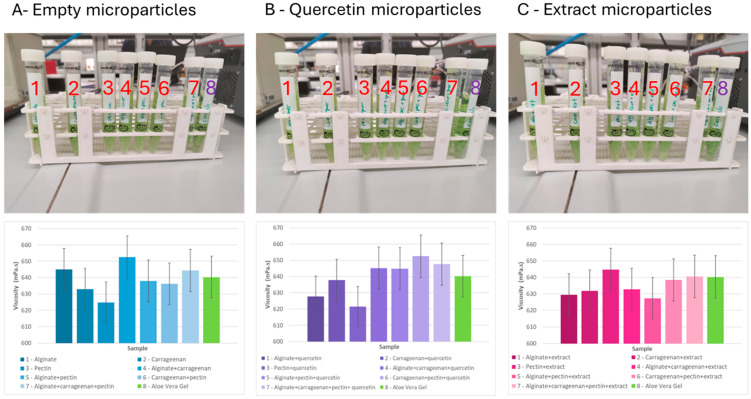
Aloe vera gel (8) fortified with different formulations of microparticles: empty microparticles and loaded with quercetin and *F. vesca* leaves extract and prepared with different encapsulating agents (alginate—1, pectin—2, and carrageenan—3) considering individual and binary/ternary blends of them (4—alginate + carrageenan, 5—alginate + pectin, 6—carrageenan + pectin, 7—alginate + carrageenan + pectin).

The microparticles were incorporated into aloe vera gel (commercial get) to obtain an amount of phenolic compound of 1 mg per 10 g of gel. The visual characterization of the samples and the viscosity evaluation of the final products were performed (Figure 5).

Comparing all the formulations, it was possible to observe that there were no visual changes in the aloe vera gel with the addition of any type of microparticles. All the samples presented the same green color, with the same degree of transparency.

In terms of viscosity, it was possible to register some changes in the values of the viscosity (gel of aloe vera viscosity: 640 mPa.s) for the different incorporations (empty microparticles: 625–653 mPa.s; quercetin microparticles: 621–653 mPa.s; extract microparticles: 627–645 mPa.s).

For the same type of microparticles, prepared with the same encapsulating agent, the inclusion of quercetin or extract had different effects in the result of the viscosity of the final formulation of aloe vera.

In general, it was possible to conclude, as expected, that the viscosity of the gel did not undergo a very significant change with the addition of microparticles.

The viscosity of the aloe vera gel in the literature can range from 90 to 10,000 mPa.s depending also on the application of the aloe vera gel [59,60,61,62]. This gel also has an interesting application in the food industry, namely in the production of edible films and coatings for food products [59,60,61].

So, the results of this work can be very promising. To mention that the use of microparticles containing strawberry (*F. vesca*) plant extract prepared with the ternary blend allowed to maintain the viscosity of the gel equal to the original formulation.

Stability tests of the same type of microparticles under different storage conditions were performed in preliminary studies and in previous works of the authors [63].

## 3. Material and Methods

### 3.1. Reagents and Solutions

*F. vesca* plants were produced in greenhouses in Vila do Conde, north of Portugal. Quercetin was used as a standard/polyphenol model (Cat. No. Q4951-100G, Lot: SLCK5305, CAS: 117-39-5) and was obtained from Sigma-Aldrich (Saint Louis, MO, USA).

For the encapsulation process, pectin, alginate, and carrageenan were used as encapsulating agents and they were purchased from Sigma-Aldrich (Saint Louis, MO, USA) with the following references: pectin (from apple); CAS number: 9000-69-5; molecular weight: 30,000–100,000 g/ mol; Cat. No.: 76,282; Lot No.: BCBN5335V (degree of esterification 70–75%; impurities ≤10% water); alginate—alginic acid sodium salt; CAS number: 9005-38-3; molecular weight: 120,000–190,000 g/moL; Cat. No.:180947-500G; Lot No: #MKBS5877V; ratio of mannuronic acid to guluronic acid (M/G ratio): 1.56, viscosity (1% H2O): 15–25 cps; carrageenan—CAS number: 9000-07-1; Cat. No.: C1013; Lot No:#SLBH9868V, moisture content: ≤12%.

For the preparation of the *F. vesca* plant extracts, ethanol at 99% was used (Cat. No. 71023001.00591), provided by Valente e Ribeiro LDA (Vila do Conde, Portugal).

For the in vitro release assays, deionized water (Milli Q water) was used, with a resistivity of 18.2 MΩ/cm at room temperature (~23 °C).

For the antioxidant activity studies were used 2.2′-Azino-bis(3-ethylbenzothiazoline-6-sulfonic acid) diammonium salt (ABTS) (MW: 548.68 g/mol), (±)-6-Hydroxy-2,5,7,8-tetramethylchromane-2-carboxylic acid (Trolox) (MW: 250.29 g/mol), and potassium persulfate and were purchased from Sigma-Aldrich (Saint Louis, MO, USA).

For the cosmetic and food incorporation studies, two commercial products were used to incorporate the microparticles: gelatine with pineapple flavor obtained from “Por Si” was used in the food test and an aloe vera gel from “Cien” (Lidl) was used in the cosmetic test.

### 3.2. F. vesca Leaves Extract Production

The production of *F. vesca* leaves extract followed the protocol optimized before by the authors [21]. Therefore, 4.0 g of *F. vesca* leaves were crushed and mixed with 80 mL of 99% ethanol and 20 mL of deionized water. The resultant mixture was stirred at room temperature for one hour at 560 rpm. After, the solution was exposed to two 30-min intervals in an ultrasonic bath (Ultrasound–Elma S30H, Elma-sonic—Singen, Germany), in a total of a one-hour treatment. The solution was centrifuged (Centromix S-549—Nogal, Spain) for 20 min, at 4000 rpm. Lastly, the supernatant was collected and stored in the freezer.

### 3.3. Microencapsulation by Spray-Drying

Three sets of solutions were prepared to feed to the spray-dryer and to produce three different types of microparticles: empty microparticles (composed only of the encapsulating agent and without bioactive compound), microparticles with only quercetin inside, and microparticles with incorporated extract.

Each set of microparticles (empty, with quercetin, and with extract) was produced with seven encapsulating agent formulations (Table 7). Thus, three of the solutions consisted of only one of the biopolymers used as an encapsulating agent (alginate, carrageenan, and pectin), another three with binary mixtures of biopolymers, and the last consisted of a ternary mixture of biopolymers.

The final concentration of the encapsulating agent solutions was 1% (*w*/*v*) and they were prepared in deionized water at room temperature and 400 rpm using a magnetic agitator (MS-H-Pro, Scansi; Portugal).

The encapsulating agent(s) solutions (individual and binary/ternary blends of biopolymers) were agitated overnight (500 rpm at ~22 °C) to enhance hydration and to help the dissolution of the wall materials.

The bioactive solutions (quercetin or extract solution) were added to the encapsulating agent solutions and mixed immediately before feeding the final solutions to the spray-dryer. The concentration of active compound in the final formulation was 2% (*w*/*w*).

A mini spray-dryer B-290 from BÜCHI (Flawil, Switzerland) with a standard 0.5 mm nozzle and a standard cyclone was used to dry the solutions and to produce the different sets of microparticles. The operational conditions of the spray-dryer were previously optimized [36]. Air pressure, aspiration rate, solution feed flow, and inlet air temperature were set at 5–6 bar, 36 m^3^/h (100%), 4 mL/min (15%), and 115 °C, respectively. The outlet air temperature varied between 47 and 61 °C.

The obtained dry powder (microparticles) was recovered and protected from the light with aluminum foil and stored at 4 °C until further analysis.

### 3.4. Scanning Electron Microscopy—SEM

According to previous studies, a scanning electron microscope (SEM, Fei Quanta 400 FEG ESEM/EDAX Pegasus X4M (Eindhoven, The Netherlands)) was used to analyze the morphology and size of the microstructures in different parts of the samples at Centro de Materiais da Universidade do Porto (CEMUP), Porto, Portugal. Different magnifications (100×–50000×) were used, and different areas of the samples were observed; the beam intensity (HV) was 15.00 kV.

### 3.5. Microparticles’ Size Distribution

All microparticles were evaluated by laser granulometry using an equipment Coulter LS 230 Particle Size Analyzer (Miami, FL, USA). Before each measurement, the microparticles were dispersed in ethanol (ethanol 99% (Valente e Ribeiro, LDA, Vila do Conde, Portugal)) to avoid the phenomena of agglomeration. The different samples were characterized in terms of number and volume size distribution. The results (mean sizes of the particles and the size distributions (in volume and number)) were obtained as an average of three runs of 30 s.

### 3.6. Conductivity of the Individual and Binary/Ternary Blends of Biopolymers

The biopolymer solutions were characterized in terms of electrical conductivity. The conductivity was evaluated with a conductivity meter (MultiLab 540, WTW—Hamburg, Germany). The conductivity analysis was performed at room temperature (~22 °C) using a 20 mL sample for each analysis.

### 3.7. Viscosity of the Individual and Binary/Ternary Blends of Biopolymers

The dynamic viscosities of the biopolymer solutions were evaluated with an Anton Paar MCR92 rheometer (Graz, Austria), with a cone-plate geometry (plate diameter of 25 mm, cone angle of 1°, and measuring gap of 0.5 mm).

### 3.8. Encapsulation Efficiency and In Vitro Release Studies

The *F. vesca* extract and quercetin release profiles of the different types of microparticles prepared were determined at room temperature, in deionized water—the simplest solvent used in industrial food and cosmetic formulations. Due to its representativeness in the extract composition, quercetin was employed as the standard compound and used to evaluate the release of the extract from the microparticles.

The release profiles were obtained using a UV–vis spectrometer (SPEC RES+, Sarspec, Porto, Portugal) by a continuous absorbance measurement (390 nm) until the value stabilized, as described by Couto and Estevinho [21].

To validate the method, a calibration curve (A = 73.66C + 0.02; A—absorbance, C—concentration) was obtained at 390 nm, using 12 standard solutions, with quercetin concentrations ranging from 0.0001 to 0.02 mg/mL. The absorbance was measured in triplicate, and its average was represented as a function of the standard concentrations. The coefficient of variation (0.99), limit of detection (LOD–0.002 mg/L), and limit of quantification (LOQ–0.006 mg/L) were determined.

In order to evaluate the kinetic behavior of the core release, two models were studied, the Korsmeyer–Peppas, Equation (1) [48], and the Baker and Lonsdale, Equation (2) [35,64,65].

(1) $$\frac{Q_{t}}{Q_{\infty }}=K_{k}t^{n}$$

(2) $$f_{1}=\frac{3}{2}\left[1-{\left(1-\frac{M_{t}}{M\infty }\right)}^{\frac{2}{3}}\right]-\frac{Mt}{M\infty }=kt$$

In Equation (1), the Qt/Q∞ represents the fraction of the active compound released until time t, while K_K_ is the Korsmeyer constant and n is the release exponent (a parameter that defines the release mechanism) [48].

Equation (2) represents the drug release from spherical monolithic dispersions, and it was developed by Baker and Lonsdale (in 1974), where the release rate constant is represented by k and corresponds to the slope. Encapsulation efficiency (%) was determined by the ratio between the mass of bioactive compound present in the microparticles and the mass of each bioactive compound used to prepare the solutions fed to the spray-dryer, considering the procedure carried out by the authors in previous works [21].

### 3.9. In Vitro Antioxidant Activity Experiment

The ABTS method was used considering adaptations made in previous works of the authors [21]. ABTS (2,2′-azino-bis-(3-ethylbenzothiazoline-6-sulfonic) acid) is an extensively used compound for determining the total antioxidant capacity of extracts of plants, food, clinical fluids, etc. This photometric assay is based on the reduction by the presence of antioxidant compounds of a well-known metastable radical (*ABTS*•+) which can be formed via numerous different approaches [66]. So, the antioxidant activity of the produced microstructures was evaluated using an ABST* radical scavenging assay. The procedure was based on the method described by Re et al., 1999 [67]. Briefly, 7.4 mM ABTS aqueous solution was prepared and mixed with 2.6 mM potassium persulfate aqueous solution and left to incubate for 16 h in the dark. After, for the preparation of the ABTS* solution, 60 mL of pure ethanol was added to 1 mL of the solution incubated overnight. The samples (150 µL) were mixed with the ABTS* solution (2850 µL) and allowed to react for 2 h, in dark conditions. The analysis was made at 736 nm using the NanoDrop One-C spectrophotometer (Thermo Fisher Scientific, Waltham, MA, USA).

### 3.10. Preliminary Experiments of Food Incorporation

The incorporation in one food product was carried out by incorporating the microparticles into a commercial pineapple-flavored gelatine (powder formulation). The gelatine was prepared according to the instructions on the package and then 20 mg of particles were dissolved in 5 mL of gelatine preparation, in order to obtain a quantity of phenolic compound of 20.4 mg per 250 mL of gelatine.

The solutions obtained were evaluated in terms of visual appearance and in terms of viscosity in the MCR 302 rheometer (Graz, Austria).

### 3.11. Preliminary Experiments of Cosmetic Incorporation

The incorporation in one cosmetic formulation was performed using a commercial aloe vera gel, where 50 mg of microparticles were dissolved in 10 g of gel in order to obtain an amount of phenolic compound of 1 mg per 10 g of gel.

The solutions obtained were also evaluated in terms of visual appearance and in terms of viscosity in the MCR 302 rheometer (Graz, Austria).

### 3.12. Statistical Analysis

All the results obtained in the present work were presented as a mean and standard deviation of the triplicates preformed for each characterization test (mean ± standard deviation). Error bars were added to the release profiles and viscosity results of the food and cosmetic incorporation. The results of statistical significance were analyzed (at a level of significance *p* ≤ 0.05) by a single-factor analysis of variance (ANOVA) and Tukey’s test, conducted by the Minitab^®^ 19 software. Excel tools were also used.

## 4. Conclusions

In this work, the microencapsulation by spray-drying of strawberry (*F. vesca*) plant extract and its main bioactive compound, quercetin, was studied. The encapsulation was carried out by using individual biopolymers, as well as binary and ternary blends of pectin, alginate, and carrageenan. Through this process, microparticles containing extract, quercetin, and empty microparticles were obtained, with an average size between 0.01 µm and 16.3 µm considering a volume size distribution and between 0.062 µm and 1.0 µm considering a number size distribution. The morphology of the microparticles depended on the formulation of the microparticle. However, for all the formulations tested, spherical microparticles with a regular shape were produced.

The encapsulation efficiency was high, varying between 81 and 100%.

The release profiles were evaluated by the Korsmeyer–Peppas and Baker–Lonsdale models, and the main release mechanism was identified as “Fickian Diffusion”, with total release times varying between 100 and 350 s.

Regarding antioxidant activity, it was possible to conclude that the microparticles that had the highest antioxidant activity were those containing quercetin.

Finally, it was possible to test the addition of microparticles in food and cosmetic products concluding that no relevant visual variations and rheological modifications were registered.

This work also proves that it is possible to conduct the valorization of agricultural by-products, creating add-value products. The extract microparticles prepared with the ternary blend showed to be one of the most promising formulations to be used for the incorporation of strawberry (*F. vesca*) plant extract in food and cosmetic products, presenting high encapsulation efficiency, a stable and well-defined release profile, and with the advantage to maintain the viscosity of the aloe vera gel and gelatine after incorporation.

In conclusion, the microparticles prepared present promising characteristics for food, nutraceutical, and cosmetic applications.

## Figures and Tables

**Table 1:** Average size of microparticles considering volume and number size differential distribution.

**Empty Microparticles**							
**Size Distribution**	**Alg**	**Carr**	**Pec**	**Alg + Carr**	**Alg + Pec**	**Carr + Pec**	**Alg + Carr + Pec**
**Volume (µm)**	4.4 ± 0.0	4.3 ± 0.0	0.3 ± 0.0	4.5 ± 0.2	0.3 ± 0.0	0.4 ± 0.0	0.2 ± 0.0
**Number (µm)**	1.00 ± 0.01	0.57 ± 0.05	0.06 ± 0.00	0.51 ± 0.17	0.07 ± 0.00	0.07 ± 0.00	0.08 ± 0.01
**Microparticles with Quercetin**							
**Size Distribution**	**Alg**	**Carr**	**Pec**	**Alg + Carr**	**Alg + Pec**	**Carr + Pec**	**Alg + Carr + Pec**
**Volume (µm)**	0.2 ± 0.0	0.1 ± 0.0	0.4 ± 0.0	0.2 ± 0.0	0.2 ± 0.0	0.2 ± 0.0	2.7 ± 0.3
**Number (µm)**	0.07 ± 0.00	0.07 ± 0.00	0.07 ± 0.00	0.09 ± 0.00	0.09 ± 0.00	0.09 ± 0.00	0.09 ± 0.01
**Microparticles with Extract of Strawberry (*Fragaria vesca*)**							
**Size Distribution**	**Alg**	**Carr**	**Pec**	**Alg + Carr**	**Alg + Pec**	**Carr + Pec**	**Alg + Carr + Pec**
**Volume (µm)**	1.5 ± 0.2	4.3 ± 0.6	10.0 ± 0.3	9.4 ± 1.3	0.2 ± 0.0	16.3 ± 2.7	4.3 ± 0.3
**Number (µm)**	0.09 ± 0.00	0.09 ± 0.00	0.36 ± 0.42	0.09 ± 0.00	0.09 ± 0.00	0.09 ± 0.00	0.09 ± 0.00

**Table 2:** Conductivity and Viscosity of the Individual and Binary/Ternary Solutions of Biopolymers.

Biopolymer Solution	Conductivity (mS/cm)	Viscosity (mPa.s)
Alg	2.27 ± 0.11	0.02 ± 0.00
Carr	3.87 ± 0.19	79.75 ± 3.99
Pec	0.01 ± 0.00	14.21 ± 0.71
Alg + carr	2.63 ± 0.13	52.74 ± 2.64
Alg + pec	1.26 ± 0.06	10.42 ± 0.52
Carr + pec	2.15 ± 0.11	59.23 ± 2.96
Alg + carr + pec	2.23 ± 0.11	50.01± 0.50

**Table 3:** Encapsulation efficiency of the different microparticles prepared.

	Encapsulation Efficiency (%)	
Encapsulating Agent Solution	Quercetin	*F. vesca* Extract
Alg	86 ± 1	92 ± 1
Carr	97 ± 1	95 ± 1
Pec	96 ± 1	86 ± 1
Alg + carr	100 ± 1	93 ± 1
Alg + pec	100 ± 1	95 ± 1
Carr + pec	81± 2	92 ± 1
Alg + carr + pec	77 ± 2	99 ± 1

**Table 4:** Korsmeyer–Peppas kinetic parameters for the different microparticles prepared.

**Microparticles with Quercetin**							
	**Alg**	**Carr**	**Pec**	**Alg + Carr**	**Alg + Pec**	**Carr + Pec**	**Alg + Carr + Pec**
**K (s^−n^)**	0.26	0.21	0.36	0.07	0.09	0.40	0.19
**n**	0.21	0.31	0.16	0.62	0.59	0.12	0.15
**R^2^**	0.80	0.77	0.74	0.70	0.85	0.70	0.62
**Microparticles with Extract**							
	**Alg**	**Carr**	**Pec**	**Alg + Carr**	**Alg + Pec**	**Carr + Pec**	**Alg + Carr + Pec**
**K (s^−n^)**	0.49	0.17	0.23	0.28	0.17	0.25	0.13
**n**	0.04	0.26	0.20	0.11	0.22	0.11	0.31
**R^2^**	0.35	0.81	0.78	0.50	0.74	0.40	0.94

**Table 5:** Baker–Lonsdale kinetic parameters for the different microparticles prepared.

**Microparticles with Quercetin**							
	**Alg**	**Carr**	**Pec**	**Alg + Carr**	**Alg + Pec**	**Carr + Pec**	**Alg + Carr + Pec**
**K (s^−1^)**	0.0028	0.0071	0.0047	0.0061	0.0061	0.0039	0.0007
**R^2^**	0.97	0.91	0.96	0.73	0.86	0.93	0.98
**Microparticles with Extract**							
	**Alg**	**Carr**	**Pec**	**Alg + carr**	**Alg + pec**	**Carr + pec**	**Alg + carr + pec**
**K (s^−1^)**	0.0020	0.0014	0.0012	0.0012	0.0012	0.0011	0.0009
**R^2^**	0.99	0.99	0.99	0.98	0.98	0.84	1.0

**Table 6:** Antioxidant evaluation of the different types of microparticles prepared.

**Empty Microparticles**							
	**Alg**	**Carr**	**Pec**	**Alg + Carr**	**Alg + Pec**	**Carr + Pec**	**Alg + Carr + Pec**
**Equivalent of Trolox (µM)**	564.6 ± 14.0	561.1 ± 9.1	563.4 ± 0.0	565.8 ± 0.0	564.6 ± 4.7	564.6 ± 4.7	564.6 ± 14.0
**Microparticles with Quercetin**							
	**Alg**	**Carr**	**Pec**	**Alg + Carr**	**Alg + Pec**	**Carr + Pec**	**Alg + Carr + Pec**
**Equivalent of Trolox (µM)**	602.4 ± 6.1	669.8 ± 69.3	623.7 ± 65.6	633.2 ± 23.8	662.7 ± 60.0	654.5 ± 9.7	627.3 ± 45.1
**Microparticles with Extract**							
	**Alg**	**Carr**	**Pec**	**Alg + Carr**	**Alg + Pec**	**Carr + Pec**	**Alg + Carr + Pec**
**Equivalent of Trolox (µM)**	583.5 ± 5.3	583.5 ± 16.0	577.6 ± 10.2	577.6 ± 10.2	572.9 ± 0.0	575.2 ± 0.0	572.9 ± 9.9

**Table 7:** Experimental design used in the preparation of the individual and binary/ternary solutions of biopolymers.

		Concentration of Each Component in the Final Solution % (*w*/*v*)		
Points	Design	Alginate (Alg)	Carrageenan (Carr)	Pectin (Pec)
1	(1, 0, 0)	1	0	0
2	(0, 1, 0)	0	1	0
3	(0, 0, 1)	0	0	1
4	(0.5, 0.5, 0)	0.5	0.5	0
5	(0, 0.5, 0.5)	0	0.5	0.5
6	(0.5, 0, 0.5)	0.5	0	0.5
7	(0.33, 0.33, 0.33)	0.33	0.33	0.33

## Data Availability

The original contributions presented in the study are included in the article; further inquiries can be directed to the corresponding author.
